# Patients with Chronic Obstructive Pulmonary Disease harbour a variation of *Haemophilus* species

**DOI:** 10.1038/s41598-018-32973-3

**Published:** 2018-10-03

**Authors:** Karen L. Osman, Johanna M. C. Jefferies, Christopher H. Woelk, Nathalie Devos, Thierry G. Pascal, Marie-Cécile Mortier, Jeanne-Marie Devaster, Tom M. A Wilkinson, David W. Cleary, Stuart C. Clarke, AERIS Study Group, J. Alnajar, R. Anderson, E. Aris, W. R. Ballou, A. Barton, S. Bourne, M. Caubet, C. Cohet, N. Coombs, V. Devine, E. Dineen, T. Elliott, R. Gladstone, S. Harden, V. Kim, S. Mesia Vela, P. Moris, K. Ostridge, M. Peeters, S. Schoonbroodt, K. J. Staples, A. Tuck, L. Welch, V. Weynants, A. P. Williams, N. Williams, M. Wojtas, S. Wootton

**Affiliations:** 1 Clinical and Experimental Sciences, Faculty of Medicine, University of Southampton, Southampton, Hants SO16 6YD UK; 2 GlaxoSmithKline Pharmaceuticals, Wavre, Belgium; 3 NIHR Southampton Respiratory Biomedical Research Unit, Southampton, United Kingdom; 4 NIHR Biomedical Research Centre, University of Southampton, Southampton, United Kingdom; 5 Wessex Investigational Sciences Hub, University of Southampton, Southampton, United Kingdom; 6 Institute for Life Sciences, University of Southampton, Southampton, United Kingdom; 7 Global Health Research Institute, University of Southampton, Southampton, United Kingdom; 8 Present Address: Merck Exploratory Science Center, Merck Research Laboratories, Cambridge, MA USA

## Abstract

*H. haemolyticus* is often misidentified as NTHi due to their close phylogenetic relationship. Differentiating between the two is important for correct identification and appropriate treatment of infective organism and to ensure any role of *H. haemolyticus* in disease is not being overlooked. Speciation however is not completely reliable by culture and PCR methods due to the loss of haemolysis by *H. haemolyticus* and the heterogeneity of NTHi. *Haemophilus* isolates from COPD as part of the AERIS study (ClinicalTrials - NCT01360398) were speciated by analysing sequence data for the presence of molecular markers. Further investigation into the genomic relationship was carried out using average nucleotide identity and phylogeny of allelic and genome alignments. Only 6.3% were identified as *H. haemolyticus*. Multiple *in silico* methods were able to distinguish *H. haemolyticus* from NTHi. However, no single gene target was found to be 100% accurate. A group of *omp2* negative NTHi were observed to be phylogenetically divergent from *H. haemolyticus* and remaining NTHi. The presence of an atypical group from a geographically and disease limited set of isolates supports the theory that the heterogeneity of NTHi may provide a genetic continuum between NTHi and *H. haemolyticus*.

## Introduction

Chronic obstructive pulmonary disease (COPD) is an irreversible, multifaceted disease resulting in degradation of lung function and is the third largest cause of global mortality^1^. Characterised by increased inflammation and mucus production in the airways alongside collapsing alveoli, the disease progresses in periods of worsening symptoms called exacerbations thought to be predominantly brought on by viral or bacterial infection^2–6^. *Haemophilus influenzae* is reportedly the most prevalent bacterial cause of exacerbations although *M. catarrhalis* has also been associated^4,5^.

*H. influenzae* is a Gram negative, opportunistic pathogen residing in the respiratory tract and occurs in encapsulated serotype (a-f) forms and non-encapsulated forms referred to as non-typeable *H. influenzae* (NTHi). Serotype b (Hib) was the largest cause of *Haemophilus* invasive disease before the introduction of the Hib vaccination, currently NTHi is the predominant cause and is also, furthermore, associated with the onset of COPD exacerbations^7–9^. Additionally, NTHi is responsible for a considerable amount of otitis media in children which can lead to chronic disease and complications resulting in hearing loss, sequelae and in rare cases, death^10^.

*Haemophilus haemolyticus* is a Gram negative commensal of the respiratory tract isolated mostly from children and COPD patients^2,11^. It is the closest relative of NTHi but is thought to be non-pathogenic. However, a small number have been reportedly isolated from invasive infection^12,13^. The two species differ in their interactions with epithelial cells, with *H. haemolyticus* resulting in cytotoxicity and NTHi, invasiveness^14,15^. Despite these differences in behaviour, the two are morphologically identical and can only be differentiated by culture due to the ability of *H. haemolyticus* to display beta haemolysis on certain agar^2,16–19^. However, this trait is not present in 100% of isolates and this has led to the misidentification of non-haemolytic *H. haemolyticus* as NTHi. A study isolating *Haemophilus* from COPD patients reported 39.5% of NTHi retrospectively re-classified as *H. haemolyticus*^2^. However, other studies have reported much lower rates of misidentification. From cystic fibrosis respiratory samples in Denmark only 0.5% were identified as *H. haemolyticus* whereas when clinical samples were retrospectively investigated 5.9% were reported as *H. haemolyticus* in Germany and 1.5% in Australia^20–22^.

The failure to distinguish between the two species by culture methods has driven the development of molecular methods. This has proven challenging due to the high levels of heterogeneity displayed within NTHi. Single, duplex or multiplex PCR assays all fail to provide 100% sensitivity or specificity in differentiating between NTHi and *H. haemolyticus*^17,23–29^. It has been suggested that the two species are therefore a genetic continuum ranging from NTHi to *H. haemolyticus*^26^. Encapsulated *H. influenzae* display lower levels of genetic diversity and are phylogenetically separable from NTHi^30–32^.

Alternatively, MALDI-TOF mass spectrometry has been recommended for differentiation between *H. influenzae* and *H. haemolyticus*; although there are conflicting reports of the ability to correctly identify the two species^33^. High levels of accuracy have been achieved due to the addition of *H. haemolyticus* and regional *H. influenzae* profiles to the MALDI-TOF database^33,34^. However, a more recent study using the MALDI-TOF bio-typer IVD 2.3 database, which includes 21 *H. haemolyticus* and 27 *H. influenzae* profiles, reported that 13.1% of *H. influenzae* and 2% *of H. haemolyticus* could not be correctly identified to species level^35^. This improved to 100% with the use of alternative newly developed software^35^. However, it should be noted that, MALDI-TOF does not identify the capsule status of a *H. influenzae* nor is there evidence of being able to distinguish the different groups.

Distinguishing *H. haemolyticus* from NTHi is important to understand the roles of both in the COPD lung, to ensure invasive or disease-causing strains of *H. haemolyticus* are not being underestimated, and to enable correct identification of the causative organism in prescribing treatment for infection. Furthermore, the question of correct taxonomical allocation *of H.influenzae* and *H. haemolyticus* and investigating how genetically related these species are is also important to further understand NTHi. The purpose of this study was to ascertain the genetic relationship between NTHi and *H. haemolyticus* isolated from COPD and answer the question surrounding current taxonomy. Here we use culture and *in silico* markers to characterise NTHi and *H. haemolyticus* from the COPD lung. Furthermore, we utilise whole genome sequencing to ascertain the phylogeny between the two species.

## Materials and Methods

### Bacterial Isolates

The Acute Exacerbation and Respiratory InfectionS in COPD (AERIS) study was a longitudinal cohort investigation of patients with moderate, severe or very severe COPD. The full study protocol has been previously detailed^5^. Sampling occurred monthly or on event of acute exacerbation spanning a two-year period and microbiological investigation was undertaken using traditional culture methods. The study was registered with ClinicalTrials (NCT01360398) and carried out in accordance with ethical approval granted by the Southampton and South West Hampshire Research Ethics Committee and in accordance with the Declaration of Helsinki and Good Clinical Practice. All participants provided written informed consent.

From the AERIS study, 1460 *H. influenzae* identified by culture, isolated from 24 patients over 134 visits were investigated^5^. Strains 4849, 7279 and 8467 of the National Collection of Type Cultures (NCTC) were used as reference isolates for *H. influenzae*. NCTC strains 10659 and 10839 were used as reference strains for *H. haemolyticus*.

### Haemolysis

All isolates of *Haemophilus* spp. were inoculated onto CBA agar supplemented with 5% horse blood (Oxoid, Basingstoke, UK) and incubated at 37 °C for 72 hours. This assay was to identify the capability of *H. haemolyticus* isolates to display beta haemolysis.

### Whole Genome Sequencing

DNA extractions were prepared from isolates cultured on chocolate agar (Oxoid, UK) using the QiaAmp minikit according to manufacturer’s instructions and diluted to 0.2 ng μl^−1^. Library preparation was carried out using the Nextera XT DNA Prep Kit (Illumina, Saffron Walden, UK). Sequencing was done using 2 × 250 paired-end V2 chemistry on an Illumina MiSeq (Illumina, Saffron Walden, UK).

### Genome Assembly and Analysis

Paired-end fastq files were trimmed of Nextera adapter sequences using trimmomatic and assembled using MaSuRCA^36,37^. Multi-locus Sequence Typing (MLST) and identification of genes for speciation was performed using SRST2^32,38^. Here paired-end fastqs were mapped to reference sequences provided in Supplementary Table 1. Resulting consensus sequences were aligned in MUSCLE^39^ prior to maximum-likelihood phylogeny analysis using RAxML^40^. Phylogenies for *omp6*, *hpd* and *smpB* were prepared using Microreact and are stored, along with associated metadata, and viewable at URLs provided in Supplementary Table 2^41^.

Further speciation was done using MetaPhlAn^42^ using the supplied 260 *H. influenzae* reference genes. Results were visualised using lattice in R (version 3.3.3)^43^. Average Nucleotide Identity (ANI) was calculated using Pyani to generate percentage genetic identity between each isolate (https://github.com/widdowquinn/pyani)^44,45^.

Core genome alignments generated in package ROARY were used to construct maximum likelihood phylogenies in RAxML^40,46^. Trees were visualised using microreact and can be accessed via http://microreact.org/project/Southampton_NTHivsHh^41^.

All MUSCLE alignments and RAxML phylogenies were completed on the CIPRES science gateway^47^. Genome assembly, MLST, gene mapping and pyani analyses were completed using the IRIDIS High Performance Computing Facility and associated IT support services at the University of Southampton.

## Results

### Initial characterisation

From the 1460 isolates of *Haemophilus* spp investigated, 12 isolates displayed haemolysis on blood agar. Further investigation demonstrated these twelve isolates could not be typed by *H.influenzae* MLST due to unrecognisable sequences at six loci and absence of the *fucK* gene. An additional 80 non-haemolytic isolates were *fucK* negative and did not have recognisable sequences at the six remaining loci. These 92 isolates were therefore classified as suspected *H. haemolyticus*. A further 54 isolates were negative for the *fucK* gene but did contain known alleles at the six remaining loci. These were categorised as *fucK* negative NTHi (f-NTHi) but were unable to be sequence typed as the MLST schema requires all seven alleles in order to allocate an ST. From the remaining 1314 isolates, 28 previously described STs were identified (n = 1076) and eight STs that were novel to this study and subsequently curated to the MLST database (STs 154, 156, 353, 356, 1314, 1441, 1442 and 1664) (n = 238). Only five STs were shared between more than one patient with ST 57, isolated from five patients, being the most prevalent.

### Molecular Markers

Raw fastq sequence data for each isolate was mapped to reference sequences (Supplementary Table 1). For genes *omp2*, *lgtC*, *fucP*, *fucK* and *iga*, presence is expected in NTHi but not in *H. haemolyticus* whereas the converse is true for *sodC*. Due to the variation displayed in NTHi of *iga* a selected sequence previously identified as the beta core and reportedly highly conserved was used for gene mapping^19^. For a further three genes, *hpd*, *smpB* and *omp6*, allelic variation distinguishes between the two species. There were unexpected results for all gene markers except *omp6* (Table 1).

**Table 1:** Presence and absence of gene markers identified by gene mapping of 1460 *Haemophilus* spp against GenBank reference sequences.

NTHi	*smpB*	*lgtC*	*iga*	*fucP*	*omp2*	*omp6*	*sodC*	*hpd*	*fucK*	Isolates with genotype (%)	No of pts (n = x/24)
G1	+	+	+	+	+	+	−	+	+	1142(83)	20
G2	+	+	+	+	−*	+	−	+	+	84(6.1)	7
G3	+	−*	+	+	+	+	−	+	+	61 (4.5)	16
G4	+	−*	+	+	−*	+	−	+	+	5 (0.4)	5
G5	+	+	+	+	+	+	−	−*	+	8(0.6)	2
G6	+	+	+	+	−*	+	−	−*	+	12(0.9)	1
G7	+	−*	+	+	−*	+	−	−*	+	1(0.1)	1
G8	+	+	+	−*	+	+	−	+	−*	53(3.9)	1
G9	+	−*	+	−*	+	+	−	+	−*	1(0.1)	1
G10	+	+	+	+	−*	+	+*	+	+	1(0.1)	1
G11	+	+	+	+	+	+	+*	+	+	1(0.1)	1
Total NTHi	1368	1301	1368	1314	1266	1368	2	1347	1314		
**Hh**	***smpB***	***lgtC***	***iga***	***fucP***	***omp2***	***omp6***	***sodC***	***hpd***	***fucK***	**Isolates with genotype (%)**	**No of pts (n = x/24)**
Hh1	+	−	−	−	−	+	+	+	−	78(84.8)	11
Hh2	−*	−	−	−	−	+	+	+	−	2(2.2)	5
Hh3	+	−	+*	−	−	+	+	+	−	12(13.0)	1
Total Hh	90	0	12	0	0	92	92	92	0		

An atypical result for the expected genotype of either NTHi or *H. haemolyticus* is denoted by *. Eleven different variations or ‘genotypes’ were identified in NTHi and three in *H. haemolyticus*. The majority 1142 (83%) of NTHi displayed the expected genotype, G1, for the molecular markers and this genotype was isolated in 20 out of the 24 patients. The majority 78 (84.8%) of *H. haemolyticus* also displayed the expected genotype Hh G1. The only molecular marker that did not result in an atypical result was that of *omp6*.

All suspected *H. haemolyticus* were negative for *omp2*, *lgtC*, *fucP* and *fucK*. However, a truncated version of the beta core sequence of the *iga* gene was unexpectedly detected in twelve *H. haemolyticus* isolates of which only two were haemolytic (Table 1). These sequences however were very short (from 100–620 bp, on average 237 bp) in comparison to the reference sequence used (864 bp) and displayed on average 93% identity to the section of the sequence. Untruncated sequences for the *iga* beta core were found ubiquitously throughout the NTHi as expected. However, *omp2* and *lgtC* were absent in 102 and 67 isolates respectively (Table 1). The 54 isolates identified as f-NTHi by MLST were also found to be *fucP* negative indicating that the entire *fuc* operon may be absent in these isolates.

For *H. haemolyticus*, as anticipated, *sodC* was found in 100% of the suspected *H. haemolyticus* but was unexpectedly observed in two NTHi (Table 1). These were isolated from two different patients and were not the same ST. Sequences for *smpB* were found in all isolates except two *H. haemolyticus* (Table 1). *hpd* was absent in 21 NTHi but present in all *H. haemolyticus*. All *hpd* negative isolates were from ST925 (n = 7) or ST819 (n = 14), furthermore, there were no *hpd* positive isolates for either ST. Similarly, the 102 isolates negative for *omp*2 were made up of ST353, ST356, ST1314 and ST819 where there were no occurrences of *omp*2 positive isolates from these STs. Some STs were found to include isolates that were both positive and negative for *omp2*. These were observed in ST11 (2 isolates out of 46), ST311(3 out of 119), ST196 (1 out of 78), ST704 (2 out of 10), ST503 (3 out of 42) and ST513 (1 out of 19). All isolates that were negative for *lgtC* were accompanied by positive *lgtC* examples within their ST. This was observed in 24 different STs and an *lgtC* absence was noted in a single f*-*NTHi isolate.

**Figure 1:**
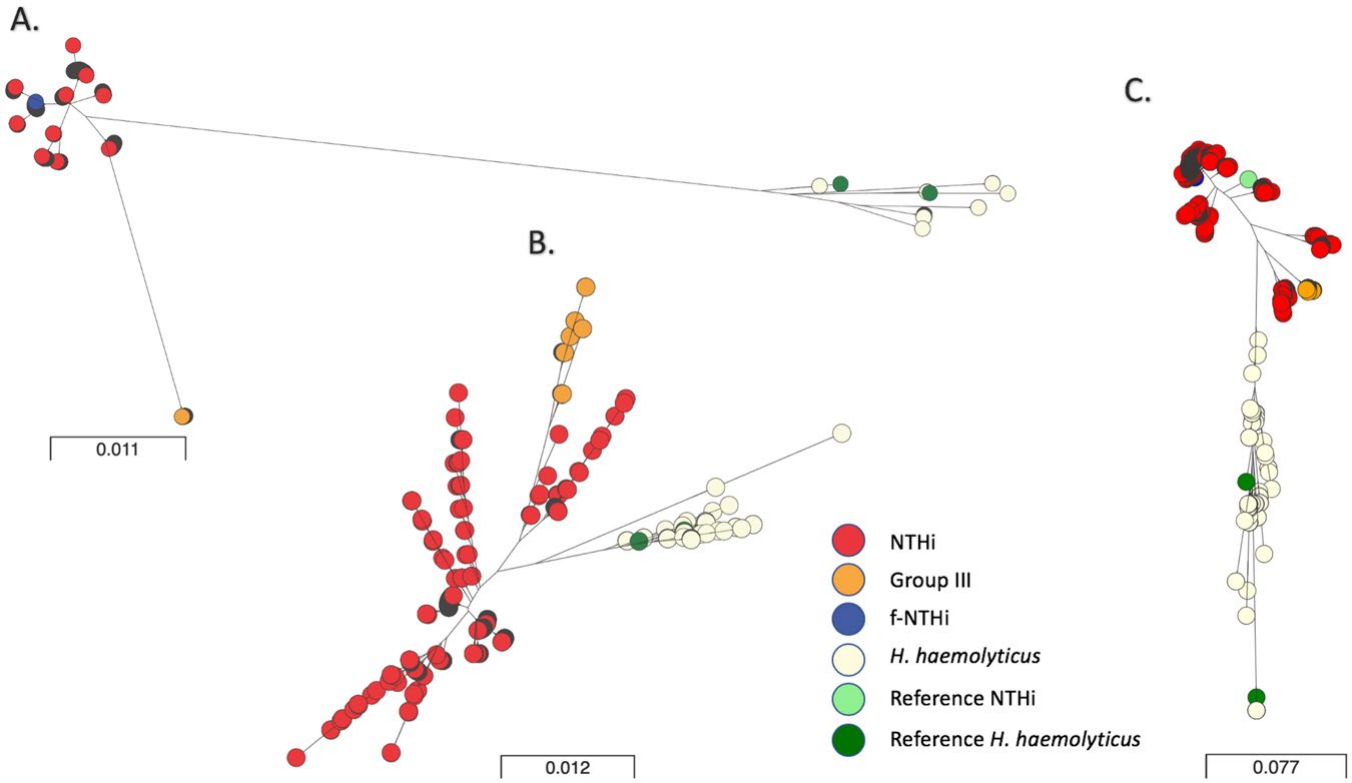
Allelic variation of (**A**) *smpB*, (**B**) *omp6* and (**C**) *hpd* from *H. influenzae* and *H. haemolyticus*. Unrooted maximum – likelihood phylogenetic trees constructed in RaxML and visualised in microreact. MUSCLE alignments of sequences for *smpB*, *omp6* and *hpd* from 1460 *Haemophilus* spp. where present using the GTRGAMMA model for nucleotide substitution. Scale bar indicates number of nucleotide substitutions per site. Colours indicate study classification group as per legend. Reference NCTC 10839 and NCTC 10659 are shown in green for *H. haemolyticus* and cluster within the *H. haemolyticus* clade. NTHi reference isolate NCTC 4842 clusters within an NTHi clade in *hpd* as marked. URL to access phylogeny and metadata available from Supplementary Table 2.

Maximum-likelihood phylogenies for *hpd*, *smpB* and *omp6* were constructed from alignments. The 92 suspected *H. haemolyticus* identified by MLST clustered into a separate lineage from all other NTHi (Fig. 1). f*-*NTHi were observed to cluster with the majority of NTHi in all three phylogenies. A group of NTHi were distinct from the others in both *hpd* and *smpB* sequence phylogenies, consisting of 76 isolates from novel STs 353, 356 and 1314, and were isolated from three different patients (Fig. 1). All were negative for *omp2*. This group was subsequently labelled as Group III and noted as a group of interest. In the phylogeny for *hpd* an adjacent clade to the Group III isolates consisted of all study isolates identified as ST513 and ST409. Also, in the phylogeny for *omp6* Group III are clustered together, adjacent on the same clade are isolates from ST513, ST311, ST704, ST 925 and ST154.

### Genetic similarity

**Figure 2:**
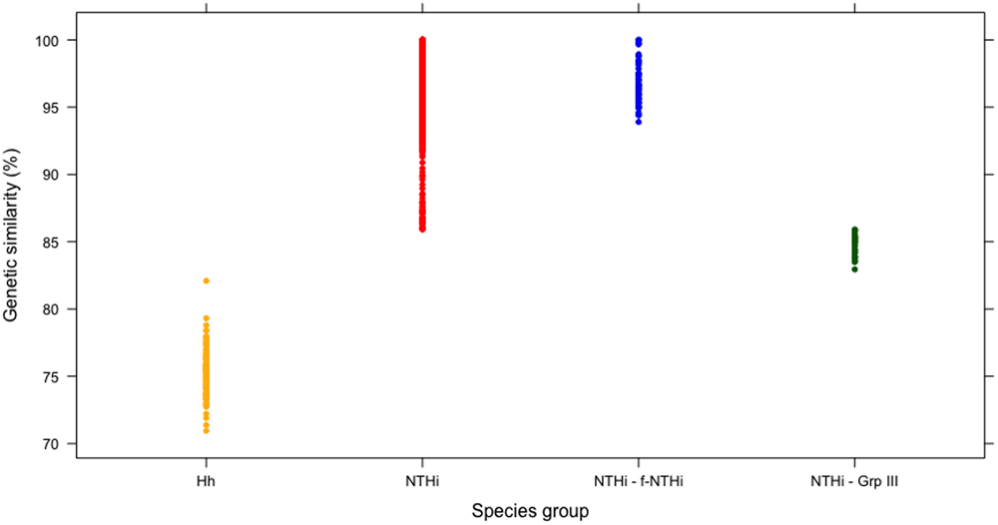
MetaPhlAn results for 1460 *Haemophilus* spp isolated from 24 COPD patients from 134 visits. Genetic similarity is based on percentage similarity of the *Haemophilus* isolates to 260 reference genes for *H. influenzae*. NTHi and f- NTHi can be seen to share a high degree of identity compared to Group III and *H. haemolyticus*.

Investigation into the genetic similarity between NTHi, f-NTHi, Group III and *H. haemolyticus* was carried out by comparing the sequence data of the isolates to the 260 *H. influenzae* reference genes held for *H. influenzae* on the MetaPhlAn database. The genetic similarity spanned from 82.93–100%. The f-NTHi were 93.89–100% genetically similar to the MetaPhlAn NTHi reference genes (Fig. 2). Group III were markedly less similar to the f-NTHi and NTHi groups with 82.94–89.97% identity (Fig. 2). All *H. haemolyticus* were found to have a much lower genetic similarity to the MetaPhlAn reference genes than the other three groups ranging from 70.94–82.09% with an average of 75.36% (Fig. 2). NTHi and f-NTHi were observed to cluster together, Group III sitting distinctly between the NTHi and *H. haemolyticus* (Fig. 2).

**Figure 3:**
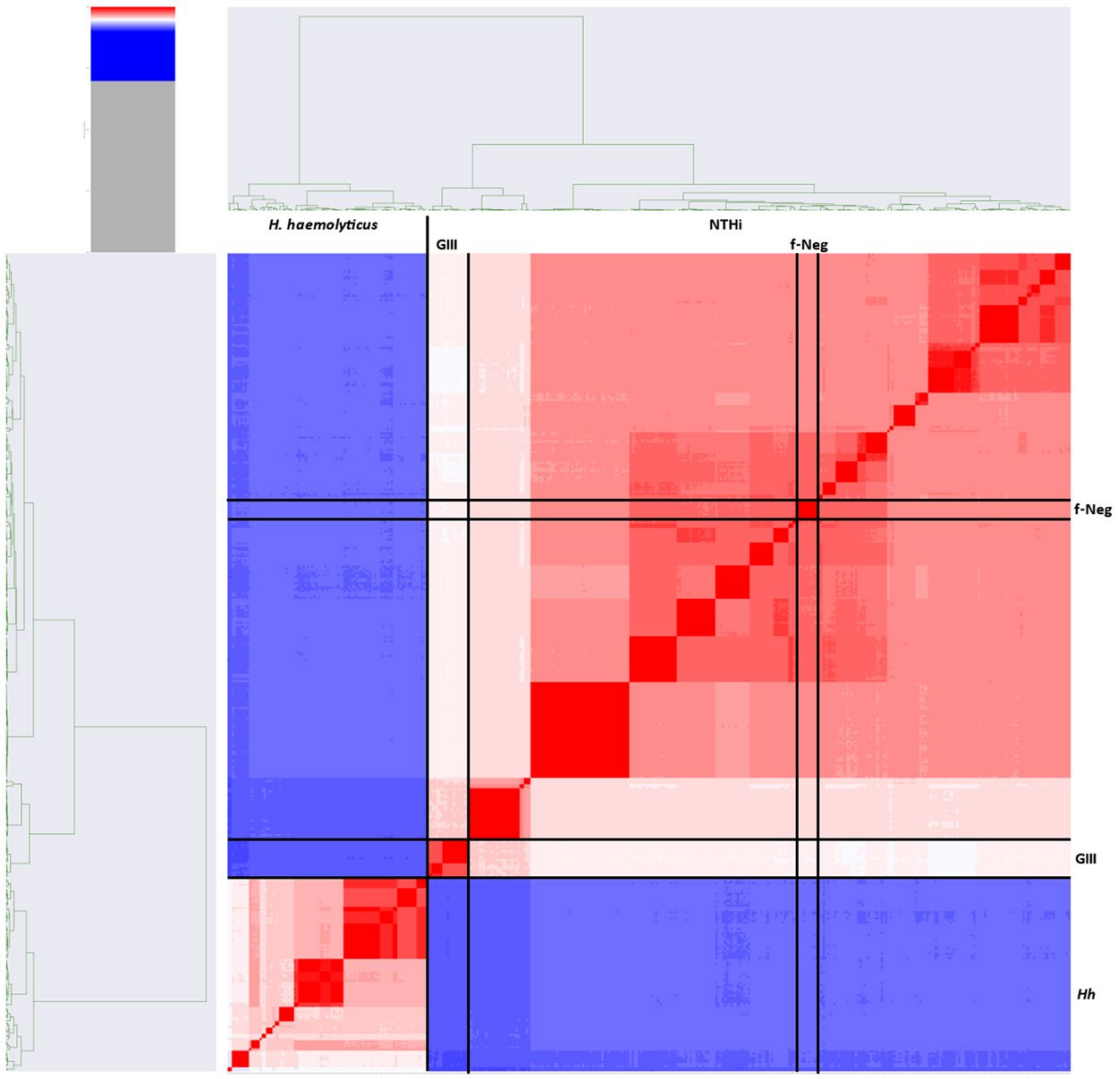
Heatmap summarising genetic similarity ANI calculations between 388 representative samples of *Haemophilus* spp isolated from 24 COPD patients over two years. Blue indicates a similarity of less than 94% and therefore a distinct species. Here this represents the *H. haemolyticus* isolates as compared to NTHi. Dark red areas depict isolates of high genetic similarity and the dendrograms show a clustering based on ST. The lighter area represents the Group III NTHi.

A subset of isolates (n = 388) was selected to represent two examples of each ST from each patient per visit alongside the suspected *H. haemolyticus*. This was done to reduce the computational complexity of the dataset for average nucleotide identity (ANI) calculation. The average genetic similarity to five reference strains was calculated for three groups; NTHi including f-NTHi, Group III and *H. haemolyticus*. The resulting heat map from ANI displays the three NTHi groups within the same species (within the red area), *H. haemolyticus* is depicted in blue indicating differentiation from the NTHi (Fig. 3). When using ANI, the criteria for inclusion as the same species dictates a genetic similarity threshold of 94–96% and above^44,48^. NTHi was observed to be within the species threshold when compared to *H*. *influenzae* NCTC reference strains 7279, 4842 and 8467 with genetic similarity averaging 96.97% ± 0.05, 96.88% ± 0.05, 96.96% ± 0.05 respectively. When compared to the *H. haemolyticus* NCTC reference strains 10659, 10839, the NTHi averaged 91.94% ± 0.008 and 91.94% ± 0.009 and therefore were differentiated from *H. haemolyticus* as this was below the species threshold (Fig. 3). Previously Group III were defined by *omp2* negative status and the distance displayed in the phylogeny in *smpB* and *hpd* from the remaining study NTHi. The ANI results further demonstrated the Group III isolates to be more divergent from the NTHi reference strains than the remaining study NTHi. The genetic similarity for Group III compared to the reference strains was on average 94.74–94.84%, noticeably less than the remaining NTHi. Using the 94% threshold for species differentiation therefore, although Group III displayed more diversity to the remaining NTHi, it is still appropriate to classify them taxonomically as NTHi and distinct from *H. haemolyticus* with the average genetic similarity at <92% between the two^44,45^. Group III therefore sits distinctly between the remaining NTHi groups and the *H. haemolyticus* (Figs 2, 3). Interestingly, in Fig. 3 the shading gradient in between the Group III and the remaining NTHi is representative of ST311, ST513 and ST704, isolates from ST513 and 311 were seen to cluster adjacent to Group III in the *hpd* phylogeny and isolates from ST513, 311 and 704 in the *omp6* phylogeny. However, these isolates displayed on average nucleotide identity of 95.2–95.5% genetic similarity to the NTHi reference strains and were *omp2* positive in the majority of cases, differentiating them from Group III.

### Core genome alignment

**Figure 4:**
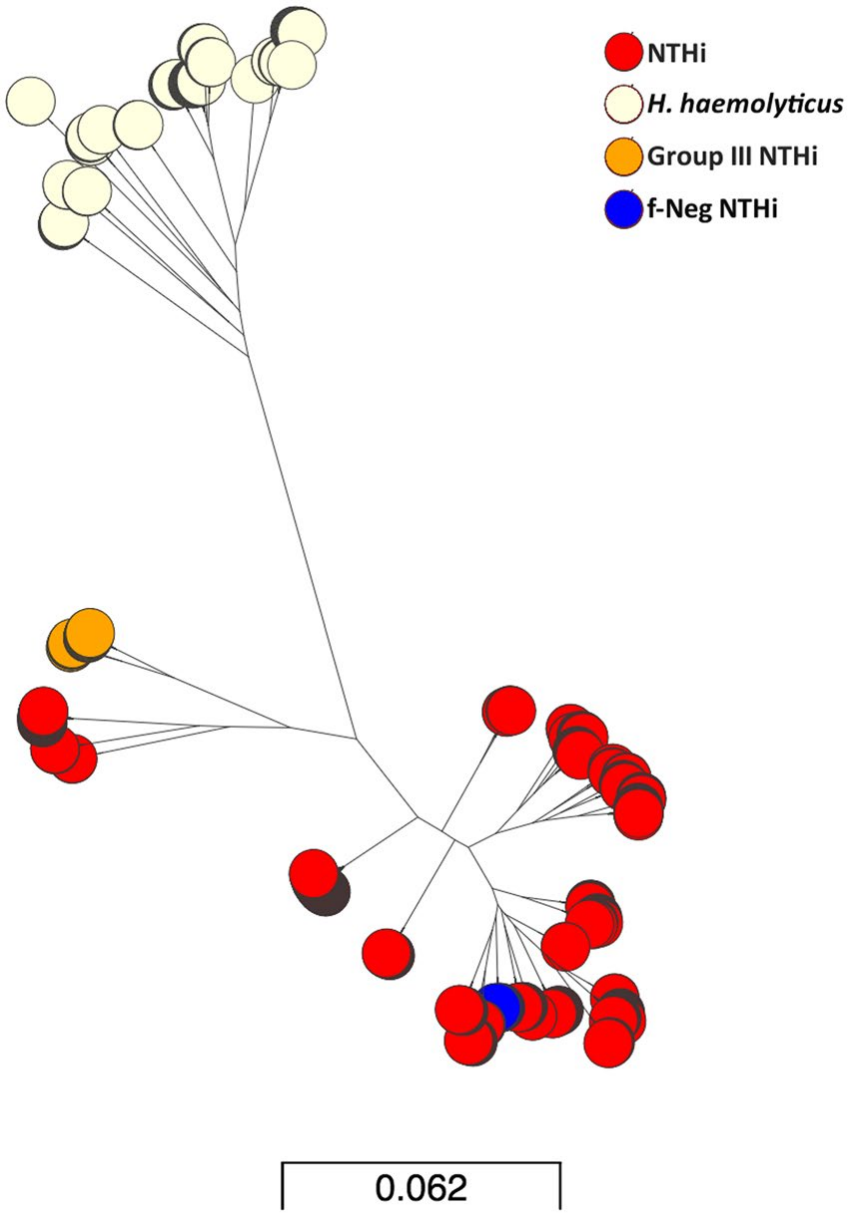
Maximum-likelihood phylogeny from core genome alignments of representative subset of 388 *Haemophilus* spp isolated from 24 COPD patients during the AERIS Study. Group III are distant from the majority of the NTHi however in this instance the Group III clade also contains NTHi STs 311, 704 and 513. The *fucK* negative isolates are observed to cluster with the majority of the NTHi and *H. haemolyticus* cluster together but distinctly from NTHi. Bar represents number of nucleotide substitutions per site. URL to access phylogeny and metadata available from Supplementary Table 2.

Phylogeny of the core genomes from the subset of isolates confirmed the population structure as identified by ANI. The *H. haemolyticus*, categorised as a separate species from all NTHi by taxonomical definition, are visibly distinct. Group III are also distinct from the remaining NTHi. All instances of f-NTHi clustered with the NTHi confirming that the absence of the *fucK* gene does not necessarily indicate a large level of diversity in the NTHi genome (Fig. 4).

## Discussion

Routine diagnostics to determine the correct causative organism in cases of infection, and in turn the appropriate prescription of antimicrobial treatment, are affected by the inability of culture methods to differentiate successfully between NTHi and *H. haemolyticus*. The ability for MALDI-TOF mass spectrometry to differentiate between the two has had success but there are cautionary tales of difficulties in correctly identifying regional strains and atypical *fucK* negative strains^33–35^. The heterogeneity of NTHi and its close genetic relationship with *H. haemolyticus* has meant the development of a successful PCR assay for differentiation has been challenging^17,26,27,29,49^ and has furthermore raised uncertainties about the species delineation between the two.

From the initial 1460 culture identified NTHi, 6.3% were confirmed to be *H. haemolyticus* and isolated from 11 patients. This ratio of *H. haemolyticus* to NTHi supports previous studies into misidentification in both clinical and study-based analyses. This does not fully support the results of a previous longitudinal COPD study that reported a 39.5% misidentification rate^2,11,20–22^. The analysis of all potential NTHi isolated from the entire AERIS study of 105 patients reported a misidentification rate of 10.6% based on lgtC/P6 duplex PCR^5^. Here we have confirmed the identity of *H. haemolyticus* by showing clear delineation between species. Furthermore, for the first time we provide evidence of a group of atypical strains that we have named Group III that show some genotypic characteristics more associated with *H. haemolyticus* and a reduced genetic similarity to typical NTHi (Figs 2, 3).

Using MLST we were able to successfully distinguish NTHi from *H. haemolyticus* as well as identify *fucK* negative isolates of NTHi. The latter represented 4% of all isolates and were isolated from the same patient over different time points. *fucP* was also observed to be absent in the f-NTHi isolates, implying the deletion of the entire operon as previously reported which raises an important question to the suitability of this loci in the MLST schema (Table 1)^50,51^. These isolates were distinguishable from *H. haemolyticus* by the presence of other identifiable alleles at the six remaining MLST loci. From the MLST analysis it was observed that only one patient carried the same ST throughout the different time points and only five STs were found in more than one patient. In total 36 different STs were identified from 24 patients. This included eight that were novel to this study and subsequently submitted for curation to the MSLT database. Three of the novel STs were characterised as the Group III (ST 353, 356, 1314). The minimal sharing of ST between patients demonstrates a large level of diversity of NTHi in this study group. Therefore, the presence of Group III isolated from three different patients and made up of three different sequence types yet displaying the same atypical genomic content is very interesting. The isolates were not only *omp2* negative but were also found to be genetically divergent to the NTHi and f-NTHi as well as closely clustering together in both cases of phylogeny of molecular markers and whole genome content. The absence of the *fucK* and *fucP* genes from f-NTHi did not predict genetic diversity from the NTHi.

Molecular markers used in isolation would have resulted in incorrect speciation which concurs with previous reports^26,50,52^. Unexpected results for species type were observed for all molecular markers that were based on presence/absence (Table 1). Recently, duplex PCR assays such as using *purT* and *hpd* or *hpd* and *fucP* have been developed. However, these have also not been found to be 100% accurate in differentiating between the two species^24,25^. The absence of more than one marker gene in some isolates was noted in this study (Table 1). This supports previously reported absence of *hpd* and *fucose* genes, and emphasizes the issues around using two molecular markers for accurate speciation^50–52^. Within this study, all markers were required to give a robust speciation. The allelic variation in *smpB* exhibited the separation of NTHi and *H. haemolyticus*; however, Group III displayed a significant diversity from the NTHi therefore interpreting the allelic information from *smpB* required supporting information. Allelic variation between species in *omp6* and *hpd* was also not deemed suitable to be used in isolation for speciation purposes. The *hpd* gene was found to be absent in 21 isolates and the diversity demonstrated by the *omp6* gene not easily interpretable between species when viewed on its own (Fig. 1). It should be noted that the *lgtC* results were questionable due to the large inconsistency of *lgtC* negative isolates with ST type in time points. This may highlight potentially false negatives due to heterogeneity within the gene, over sensitivity of the mapping method or level of gene coverage in the sequencing data which would require further phenotypic investigation or traditional PCR to ascertain gene presence. However, there were no *lgtC* occurrences identified in *H. haemolyticus*. Additionally, it is very doubtful that the *iga* beta core sequences identified in twelve *H. haemolyticus* would be instrumental in Iga protein expression, the identified sequences were on average around 236 bp long compared to the 864 bp long sequence for the beta core which in itself is a selected sequence, reportedly conserved, from a much larger gene^19^. This again could be a result of over sensitivity or previously highlighted limitations of the mapping method.

Surface exposed proteins resulting from some of the genes discussed in this study, Protein D, Omp2 and Omp6, have also been investigated as potential vaccine candidates^52–54^. The importance for vaccination for NTHi does not come from its ability to cause invasive disease but is driven by the larger morbidity caused by NTHi in cases of otitis media and the role NTHi plays in triggering exacerbations in COPD. Figures differ globally but it is thought that 80% of children will suffer from acute OM before the age of five^10^. In the UK in 2016, the prevalence of OM among children under the age of five was reported as 2469.5 per 100,000^1^. COPD in the UK is reported to have attributed to 6.4% of all deaths in 2016^1^. A vaccination for NTHi, therefore, could result in a reduction of disease and associated economic burden. However, designing a vaccine for a subspecies as heterogeneic as NTHi has not proven straight forward. Changes in protein expression mediated by sequence variation, genotype absence and phase variation all hinder the discovery of a successful candidate. The absence of *omp2* and, to a lesser extend *hpd*, from NTHi, both in this study and in previous reports, raises the question of the validity of these targets for vaccine candidature^26,52^. The sequence variation throughout *omp6* demonstrated within this study supports other previous reports and may result in variability of exposed regions and thus, potentially, a risk to vaccine efficacy (Fig. 1)^55^.

It has been suggested that *H. haemolyticus* and NTHi are two ends of a genetic continuum of one species^26^. Using whole genome analysis, the isolates in this study fell into three groups (NTHi including f-NTHi, Group III and *H. haemolyticus*) rather than a continuation of genome diversity but this does consider the limitations of this study derived from bias drawn from limited isolation site and disease, and limited geographical area of patient recruitment. *H. haemolyticus* was distinguishable from NTHi in phylogeny for *hpd*, *smpB* and *omp6* sequences and identified as a different species from NTHi using ANI, which has been deemed comparable for taxonomy purposes to the gold standard: DNA-DNA hybridization (Fig. 2)^45^. Group III were calculated as approximately 92% genetically similar to *H. haemolyticus*, a similar divergence from *H. haemolyticus* to the remaining NTHi (Figs 3, 4). However, ANI is only one taxonomy tool and does not consider orthologous content of the genomes, it also relies on alignment for comparison which may disregard some of the genome; however, it gives a good indication of the phylogenetic relationship between strains.

## Conclusion

In this study, *H. haemolyticus* and NTHi are definable as two species, but the presence of Group III demonstrates the diversity of NTHi in a limited study set. These isolates are genetically divergent from NTHi, sit phylogenetically between the remaining NTHi and *H. haemolyticus* and display an unexpected genotype for NTHi. Group III represent a lineage of atypical strains composed of three novel STs (353, 356, 1314) that were identified from three separate patients. With the addition of genomic data from NTHi isolated from different geographical locations, disease states and carriage samples, other examples of differing atypical groups may occur and further muddy the boundaries between the two species. Interestingly though, genetically the similarity to *H. haemolyticus* was no greater than NTHi or f-NTHi. Hypothetically, Group III may represent a lineage that requires further investigation to ascertain whether survival techniques have developed in these isolates specific to the COPD niche. Further research is also required to understand their potential in colonisation and virulence.

The misidentification rate of *H. haemolyticus* as NTHi was relatively low in this study echoing previous studies investigating this matter^20–22^. As previously reported, further investigation required to confidently distinguish the two species may not warrant the time consuming and expensive assays when faced with the need for timely treatment for potential *Haemophilus* infection^20–22^. Ultimately the heterogeneity in NTHi leaves single molecular markers insufficient to delineate with 100% accuracy.

## Electronic supplementary material

Supplementary material

## Data Availability

The results summary for this study (GSK study number 114378 NCT 01360398) is available on the GSK Clinical Study Register and can be accessed at www.gsk-clinicalstudyregister.com. For interventional studies that evaluate our medicines, anonymized patient-level data will be made available to independent researchers, subject to review by an independent panel, at www.clinicalstudydatarequest.com within six months of publication. To protect the privacy of patients and individuals involved in our studies, GSK does not publicly disclose patient- level data.
