# Supplementary material for: Patients with Chronic Obstructive Pulmonary Disease harbour a variation of Haemophilus species
Source: Sci Rep. 2018 Oct 3;8:14734. doi: 10.1038/s41598-018-32973-3 (PMC6170463; doi:10.1038/s41598-018-32973-3)
Supplement: Supplementary file 1 — Supplementary material [file 41598_2018_32973_MOESM1_ESM.pdf]

Patients with Chronic Obstructive Pulmonary Disease harbour a variation of *Haemophilus* species.

Authors:

Karen L. Osman[1], Johanna M.C. Jefferies[1], Christopher H. Woelk[1#], Nathalie Devos[2], Thierry G. Pascal[2], Marie-Cécile Mortier[2], Jeanne-Marie Devaster[2], Tom M.A Wilkinson[1,3,5], David W. Cleary[1,3 ##], Stuart C. Clarke[1,4,5,6,7 *##]

1. Clinical and Experimental Sciences, Faculty of Medicine, University of Southampton, Southampton, Hants SO16 6YD, UK.
2. GlaxoSmithKline Pharmaceuticals, Wavre, Belgium
3. NIHR Southampton Respiratory Biomedical Research Unit, Southampton, United Kingdom.
4. NIHR Biomedical Research Centre, University of Southampton, Southampton
5. Wessex Investigational Sciences Hub, University of Southampton, Southampton
6. Institute for Life Sciences, University of Southampton, Southampton
7. Global Health Research Institute, University of Southampton, Southampton, United Kingdom

* Corresponding author S.C.Clarke@soton.ac.uk

# Current affiliation: Merck Exploratory Science Center, Merck Research Laboratories, Cambridge MA, USA.

Joint last authors.

Supplementary material.

Supplementary table 1. Accession numbers for GenBank reference sequences used for mapping.

Gene	Genbank Accession
omp2	CP000057.2 (216249…217846)
omp6	M19391
fucK	CP009610.1 (287354..288766)
fucP	CP000057.2 (823471…824757)
hpd (*glpQ*)	CP002277.1 (1826771…1827865)
iga	M87492 (4124…4978)
lgtC	CP002277.1 (315813…316835)
smpB	NC_000907.1 (1038488…1038973)
sodC	AFQQ01000001.1 (155446…156018)

Supplementary table 2. URLs to access Microreact phylogeny and metadata

Phylogeny	Microreact URL
omp6	https://microreact.org/project/BkT-nmKsM
hpd (*glpQ*)	https://microreact.org/project/SkerJeLrZ
smpB	https://microreact.org/project/HJtpSwaGW
core genome	https://microreact.org/project/SJS9PP7LW
